# Supplementary material for: Limiting spread of COVID-19 in Ghana: Compliance audit of selected transportation stations in the Greater Accra region of Ghana
Source: PLoS One. 2020 Sep 11;15(9):e0238971. doi: 10.1371/journal.pone.0238971 (PMC7485755; doi:10.1371/journal.pone.0238971)
Supplement: S1 File — (DOCX) [file pone.0238971.s001.docx]

**LORRY STATION HYGIENE AND SOCIAL DISTANCING ASSESSMENT TOOL**

**Study title:**

**IS GHANA PREPAREDFOR COVID 19? AN ASSESSMENT OF SELECTED LORRY STATIONS IN ACCRA**

**h**

| Name of Lorry Station | …………………………………………… |
| --- | --- |
| Station Type | Mini buses [ ]  Taxi [ ]  Long buses [ ] |
| Name of locality/area where lorry station is located | …………………………………………... |
| Municipality/District | ……………………………………………. |
| Name of person assessing the station | ………………………………………….... |
| Date of Assessment | …………………………………………… |

| Expected Observation Times | **8am-10am**  **12am-2pm**  **3pm-5pm** |
| --- | --- |
| Minimum Expected Observation Time at a Station | **30 minutes** |
| Start time: |  |
| End time: |  |

| **SECTION A. PERSONAL HYGIENE REMINDERS/ANNOUNCEMENT** | | | |
| --- | --- | --- | --- |
| **NO.** | **Observational Question** | **Response** [ tick only one] | **Score** |
| 1 | Are posters with information on hand hygiene or locally produced equivalent with similar contentdisplayed in the lorry station? | Not displayed [ ] | 0 |
|  |  | Displayed in some areas [ ] | 5 |
|  |  | Displayed in most areas [ ] | 10 |
|  |  | Displayed in all areas. [ ] | 15 |
| 2 | Are there posters showing/explaining the correct hand washing techniques? | Not displayed [ ] | 0 |
|  |  | Displayed in some areas [ ] | 5 |
|  |  | Displayed in most areas [ ] | 10 |
|  |  | Displayed in all areas. [ ] | 15 |
| 3 | Are other hygiene reminders (e.g. coughing or sneezing into tissue paper/elbow) available? | Not displayed [ ] | 0 |
|  |  | Displayed in some areas [ ] | 5 |
|  |  | Displayed in most areas [ ] | 10 |
|  |  | Displayed in all areas. [ ] | 15 |
| 4 | Are there announcements about hand/personal hygiene at the lorry station? | No announcement at all [ ] | 0 |
|  |  | Announcement made only once [ ] | 5 |
|  |  | Announcement made severally [ ] | 10 |
|  |  | Announcement made all the time [ ] | 15 |
| **SECTION B. AVAILABILITY OF HYGIENE FACILITIES** | | | |
| 5 | Is there a place for hand washing at the lorry station? | Yes [ ] | 10 |
|  |  | No[ ] | 0 |
| 6 | If there is a place for handwashing, how many? | Only 1 [ ] | 5 |
|  |  | More than 1 [ ] | 10 |
| 7 | Nature of hand washing place? | Sink only[ ] | \| 10 \| \| --- \| |
|  |  | Veronica Bucketonly [ ] | 10 |
|  |  | Sink and Veronica bucket | 20 |
|  |  | Other[ ]  Please, specify………………………  ……………………………………………………………………………… | 10 |
| 8 | Is/are there handwashing place(s) available to all at the station? | Yes [ ] | 10 |
|  |  | No[ ] | 0 |
| **SECTION C. AVAILABILITY OF WATER** | | | |
| 9 | Is water available for hand washing? | Yes [ ] | 20 |
|  |  | No[ ] | 0 |
| 10 | If water is available, is it running tap water, Veronica Bucket or water stored in a container? | Running tap water[ ]  Veronica Bucket[ ]  Water Stored in a container[ ] | 5  5  5 |
| 11 | If water is available, is it visibly clean? | Yes [ ] | 5 |
|  |  | No[ ] | 0 |
| **SECTION D. AVAILABILITY OF DETERGENTS** | | | |
| 12 | Is soap (liquid/solid) available at the hand washing place? | Yes [ ] | 10 |
|  |  | No [ ] | 0 |
| 13 | Is alcohol-based hand sanitizer available at the lorry station? | Not available at all [ ]  Available at only one point [ ]  Available at more than onepoints [ ] | 0  5  10 |

| **SECTION E. USE OF HAND WASHING FACILITIES& HAND SANITIZER** | | | |
| --- | --- | --- | --- |
| 14 | Are the handwashing facilities being used frequently, infrequently or not at all? | Frequently used[ ] | 10 |
|  |  | Infrequently used[ ] | 5 |
|  |  | Not used at all [ ] | 0 |
| 15 | Are there people in a queue waiting to wash hands? | Yes [ ] | 5 |
|  |  | No[ ] | 0 |
| 16 | Are people using soap when they wash hands? | Yes [ ] | \| 10 \| \| --- \| |
|  |  | No[ ] | 0 |
| 17 | Are people using alcohol-based hand sanitizer when they board/unboard buses/cars? | Yes [ ] | 10 |
|  |  | No[ ] | 0 |
| **SECTION F. SOCIAL DISTANCING** | | | |
| 18 | Any visible/recognizable communication/messages on social distancing, *eg poster/audio message,etc* | Yes [ ] | 5 |
|  |  | No[ ] | 0 |
| 19 | Any infrastructural or spatial changes to ensure social distancing *eg barricades for how to stand in queues, etc* | Yes [ ] | 5 |
|  |  | No[ ] | 0 |
| 20 | Any arrangements by the lorry station drivers/mates/leaders to promote social distancing *eg queuing, boarding or seating arrangements* | Yes [ ] | 5 |
|  |  | No[ ] | 0 |
| 21 | Any observable actions by passengers to observe social distancing from other passengers within lorry station?  *Eg deliberate individual attempts to maintain social distancing* | Yes [ ] | 5 |
|  |  | No[ ] | 0 |
| 22 | Any observable actions by other persons in the lorry station (including vendors, load bearers, etc) to observe social distance when interacting with passengers? *Eg attempts to maintain a distance with passengers or their goods* | Yes [ ] | 5 |
|  |  | No[ ] | 0 |
| 23 | Wearing of protective clothing/equipment (PPE’s), *eg nose mask or other similar PPE’s* | Not worn at all [ ]  Worn by a few[ ]  Worn by many[ ]  Work by all [ ] | 0  5  10  15 |
| 24 | Are passengers making an effort not to touch surfaces that can lead to spread of the virus eg car doors, seats, station chairs, etc? | Yes [ ] | 5 |
|  |  | No[ ] | 0 |
| 25 | Are passengers seen making an effort to keep social distance from vendors in the station? | Yes [ ] | 5 |
|  |  | No[ ] | 0 |
| 26 | Are passengers seen making an effort to keep social distance from mates/drivers? | Yes [ ] | 5 |
|  |  | No[ ] | 0 |

OTHER OBSERVATIONS (Please, provide as much detail as possible)

……………………………………………………………………………………………………………………………………………………………………………………………………………………………………………………………………………………………………………………………………………………………………………………………………………………………………………………………………………………………………………………………………………………………………………………………………………………………………………………………………………………………………………………………………………………………………………………………
