# Supplementary material for: Limiting spread of COVID-19 in Ghana: Compliance audit of selected transportation stations in the Greater Accra region of Ghana
Source: PLoS One. 2020 Sep 11;15(9):e0238971. doi: 10.1371/journal.pone.0238971 (PMC7485755; doi:10.1371/journal.pone.0238971)
Supplement: Attachment — Submitted filename: Response to Reviewers Aug 7.docx [file pone.0238971.s003.docx]

**REBUTTAL LETTER**

**PONE-D-20-13058
Limiting Spread of COVID-19 in Ghana: Compliance audit of selected transportation stations in the Greater Accra region of Ghana**

**RESPONSES TO ACADEMIC EDITOR’S COMMENTS**

**Comment**

Please provide more information of the selection criteria used to choose the 45 transport stations in this study, along with the names of the 45 transport stations.

**Response –Page 6/7, lines 123- 137**

“The GAR has a population of almost five million with over 137 registered market centers and their corresponding public transportation stations[15]. There are 2 metropolises, 9 municipalities and 5 districts, making a total of 16 administrative units in the GAR. These administrative units differ significantly in size, and volume of business activities, which is usually highest at the metropolitan level and lowest in a district.

To ensure that our sample reflects this heterogeneity in the GAR, of the 16 administrative units in the GAR, 11 were included in the study. They included two metropolitan cities (Accra and Tema), seven municipal cities (Ashaiman, Kpone-Katamanso, La Nkwantanang Madina, Ayawaso

West, Ablekuma North, Ablekuma South, and Ga East) and two districts (Ningo-Prampram, and Ga West)

There is	no existing documented evidence of daily passenger traffic across stations in the country. Within each of the selected units, based on the judgement of the authors, the lorry stations that are generally noted to carry a relatively high daily passenger traffic	in the intra- city transportation sector were selected.

The names of the mini bus stations were; Amasaman Main Station, Ashaiman-main, Atadeka-Madina, Ashaiman-Zenu, Tema Station, Prampram Main Staion, Kwabenya Trotro Station, Tudu-Inter-City, Haatso ECOMOG Lorry Station, Atadeka-Ashaiman, Dome Trotro Crossing, Achimota Terminal, Madina Lorry Station, Pokuase-Ayawaso Branch	Tudu-Main, Abokobi , Agbogbloshie, Madina Old Road, Legon Trotro Station, 37, and Old Ashongman lorry stations.

The taxi stations were Ashaiman-Jericho, Amasaman Main Station, Prampram Last Stop, Amamolye-Pokuase, Estate Junction Taxi Station Community 11 and 12, Dawhenya , Community 5 and 6, Abokobi , Atadeka-New York, Dome Taxi Crossing, Market square, Methodist Junction , Festus-Station, Legon , Haatso ECOMOG , Community 1 and (5 and 6) taxi stations.

The third group of stations were for long buses. They are Neoplan, OA, VIP, STC Accra, Ayalolo Terminal, Great Empirial, and VVIP stations.

“The names of the lorry stations are listed in Fig1”- Page 7, line 139-140

**Comment**

Please remove "2. There was an anticipated lock-down by the Government that made it impractical to either submit an ethics application or carry out the study as planned without going to the field, immediately. Considering there will be no human interaction and the methods do not pose any harm to individuals, authors, therefore judged it ethically appropriate to proceed in order not to lose the opportunity for implementing the study. The decision was also strengthen by the lack of information on when the lock down will be lifted" from the ethics section of your manuscript, as this is not a valid reason in this case to not obtain ethics approval.

**Response-page line**

The paragraph has been deleted from the submission system as requested

**Comment**

We note that you have indicated that data from this study are available upon request. PLOS ta to be available upon request if there are legal or ethical restrictions on sharing data publicly. For information on unacceptable data access restrictions, please see <http://journals.plos.org/plosone/s/data-availability#loc-unacceptable-data-access-restrictions>.

In your revised cover letter, please address the following prompts:

a)If there are ethical or legal restrictions on sharing a de-identified data set, please explain them in detail (e.g., data contain potentially identifying or sensitive patient information) and who has imposed them (e.g., an ethics committee). Please also provide contact information for a data access committee, ethics committee, or other institutional body to which data requests may be sent.

b) If there are no restrictions, please upload the minimal anonymized data set necessary to replicate your study findings as either Supporting Information files or to a stable, public repository and provide us with the relevant URLs, DOIs, or accession numbers. Please see <http://www.bmj.com/content/340/bmj.c181.long> for guidelines on how to de-identify and prepare clinical data for publication. For a list of acceptable repositories, please see <http://journals.plos.org/plosone/s/data-availability#loc-recommended-repositories>.

**Response**

There are no restrictions on the dataset and we have uploaded the dataset in a publicly accessible data repository. The name of the repository is Figshare and the digital object identifiers (DOI) is 10.6084/m9.figshare.12644897. Access the data using the private link: <https://figshare.com/s/a0b1076e14a002071911>

**RESPONSES TO REVIEWER 1 COMMENTS**

**Comment-abstract**

How many washing places per station should be adequate?

**Response: Page page 20, line 380-381**

The existing literature on adequacy of and Water, Sanitation and Hygiene facilities (WASH ) facilities in out of home settings (mainly Schools and Health facilities) do not provide standards on adequate number of hand washing stations for transportation stations. We anticipate that if majority of commuters were to follow government hand hygiene recommendations for limiting COVID-19 transmission, having only one washing basin will not be sufficient, given the large number of public transportation users in the city of Accra. Further research will be needed to understand what constitutes adequate washing places per transportation station.

**Please refer to Page page 20, line 380-381**

“Studies are needed to determine standards on how many washing places are needed in public places like transport stations.”-

Studies are needed to determine standards on how many washing places are needed in public places like transport stations.

**Comment –abstract-**

Were there any other type of hand sanitizer beside alcohol based ones?

**Response- Page 14, line 256-258**

Our aim was to measure compliance with the preventive measures introduced by the Ghanaian National COVID-19 Response team to limit the spread of the disease. The recommendations specify regular rubbing of hands with alcohol based sanitizers among others. Therefore, we did not capture the availability and use of other non- recommended sanitizers, aside alcohol based sanitizers. Please refer to the data collection tool **(Section D & E, questions; 12- 17).**

We have addressed this concern in **Page 14, line 256-258**

“We did not observe the availability and use of other types of sanitizers, apart from alcohol-based sanitizers which had been recommended by the government”

**Comment**

The authors have indicated that no approval was sought for this observational study because 1) there was no direct contact with human subjects, and 2) an anticipated lock-down of the country. The Helsinki Declaration of 1975, revised in 2000, which is an ethical standards for international committee of medical journal editors, stipulates that authors are mandated to indicate whether the procedure employed follows ethical standards by institutional/ national ethical review boards. The lack of human contact in this study does not exempt the study from ethical review since the Helsinki Declaration is applicable to all types of studies including observational ones.

Even though laws regarding the conduct of biomedical research vary country by country, the reasons given by the authors does not absolve them from seeking approval. Ethical review boards both institutional and national, knowing the gravity of Covid-19 pandemic. Instituted expedited reviews for covid-19 related research to allow researchers to share current information on the pandemic. Authors should take advantage and obtained an expedited review or exemption letter from the IRB/Health Ministry.

**Response**

The research qualified for exempt from ethics review because: 1) the study was planned for implementation in a public space where there is no expectation of privacy; and 2) the study procedures does not require direct interaction with human subjects and no individual personal identifiers have been reported in the manuscript. However, the authors were unable to submit the protocol to the IRB for a determination of ethics exemption, as required, because most institutions were preparing for a lockdown of the city and the IRB was not receiving ethics applications for review during that period.

**Comment**

The authors indicated the study had no human interactions, just observations so what specific data sets were obtained that could have legal implications and ethical concerns bearing in mind no ethical approval was even sought for the study?

It is best practice for authors to deposit their dataset in institutional/public repositories. This is very important because greater number of emails to corresponding authors that hold study datasets mostly do not get response after publication.

**Response**

There are no identifying information or behaviors that can be linked to individuals in this dataset. As such the dataset has been made publicly available at Figshare and the digital object identifier (DOI) is 10.6084/m9.figshare.12644897

**Comment-10**

Repetition at line 165 to 166 and line 171-172

**Response**

The noted repetition at Line has been deleted. Please check page 9, line 181-183

**Comment-11**

Aside wearing nose mask/face shield (uncommon), is that other PPE that passengers could wear?

**Response-Page 14, line 268-269**

At the time of data collection, the government had not mandated the use of PPE in the country.

“We observed the use of handkerchiefs, headgears, and personal clothing being used as face masks.”

**Comment 12 – page 10, line 202 to 203**

Table 1. Separate or section such that station type and District/municipality do not align because the table in its current state makes the frequency totals 90 not 45

**Response page 11, line 226-227**

Marginal totals have been used to separate the station type and District/municipality as recommended.

**Comment 13 –page 12, lines 231 to 232**

Were the situations where passengers used their personal hand sanitizers other than those provided at the stations?

**Response –page 21, line 386- 388**

Our study was designed to observe the availability of hygiene facilities at lorry stations and whether these facilities were used by passengers at the station. Thus, data was not collected on the use of personal sanitizers by passengers.

We have provided further information in the discussion section to address this concern.

“It is possible that some passengers may have used their own personal alcohol-based sanitizers before boarding or alighting from vehicles. However, the study was not designed to measure this behavior”.- **page 21, line 384- 386**

**Comment – page 16 and 17, line 297 to 299**

The idea to implement best and routine public safety standards is very laudable but how will your proposed air travel measures be achieved considering infrastructural limitations in these stations?

**Response- page 20, line 367-379**

Despite these limitations, the government has already put out regulations to implement some airline-industry type travel measures as described on **page 20, line 367-379.** To be sustainable, these travel measures should be contextualized within the local situation, so that they can be enforced without compromising their effect.

**Comment 15 -page 17, line 308 to 310**

What actionable steps do the authors propose/recommend that transportation services adopt to safeguard passengers?

**Response- Page 20, line 367-379**

Specific actions that can contribute to addressing the guidelines adherence gaps observed in the current study include enforcing mandatory wearing of face masks including both transport operators and travelers, frequently cleaning surfaces including door knobs, seats, counter tops, and arm rests of vehicles using appropriate sanitizing agents. Enforcing mandatory hand hygiene will only work if the government, at all levels provides financial incentives and technical support for transport operators to install and enforce use of handwashing and hand sanitizing infrastructure. In addition to these, interventions to ensure appropriate physical distancing and implementation of other relevant guidelines when vehicles are in transit are warranted. Presently, use of face masks is mandated by City of Accra regulations. Specific physical distancing on board public transportation was also established early in the pandemic. The main challenge to implementing these recommended actions will be enforcement. Considering the observed poor adherence to the existing guidelines observed in the transport stations, further research is needed to understand the drivers of behavior change related to these guidelines.

**Comment-16 –page 17, line 316**

Rephrase “especially at the at lorry stations”

**Response –page 18, line 337**

The phrase has been rephrased. It reads as follows: “especially at the lorry stations”

**Comment 17 -page 18, line 325**

Replace There with “They”

**Response –page19, line 346**

“There” has been replaced with” they” as requested.

**Comment – 18 Page 18, line 341 to 343**

Why is the use of face mask in crowded places a health concern? Should it not rather be non-use of face masks?

**Response-page 19, line 359-361**

The sentence has been reworded. It reads as follows: “The non-use of face masks in crowded transportation stations, where the practice of social distancing is almost non-existent is a public health concern”

**Comment -19 – pages 19-20, line 357 to 367**

What interventions have been taken by the authors based on the study findings at the transport stations and operators? Are they aware of the study outcome knowing they are not privy to publication of the findings in scientific journals? Any education on covid-19 related risks and preventive measures on-going?

**Response**

A research brief was shared with the National COVID-19 response team in early April 2020 to guide decision making, especially in the transportation sector. In addition, members of the research team have been sharing the study findings and recommendations in multiple electronic and print media.

**Comment-**

The authors, however, failed to outline the needed stringent preventive measures that transportation managers/owners could adopt at the stations to enhance compliance in the discussion.

**Response –Page 20 line 367-381**

Thanks for your response. Kindly refer to Page 20, line 367-379 for specific actions to address the risks of COVID-19 transmission

**RESPONSES TO REVIEWER 2 COMMENTS**

**Reviewer #2:**

The manuscript is neither technically sound nor add something novel to the literature. Nothing has been done to compensate for the individual bias that could be introduced during the collection process. Also, no correlation has been made between the numbers of cases that have been reported at that time in each of the area that has been mentioned.

**Response**

There are no comments in the attached PDF file to help the authors to understand how the reviewer’s conclusion was arrived at. We humbly request the reviewer to kindly provide additional information to help the authors understand what the gaps are that makes the manuscript not technically sound.

To ensure repeatable observations and minimal inter-observer differences a tool that requires simple observations was designed and pretested prior to data collection. Analyses of the pretest results showed that the observations reported by individual observers were reproducible and valid. We have further described these processes in the methods section, **page 7, line 144-145, and page 8, line 159-164**

The study was designed to understand how preventive guidelines were being adhered to. Thus, it was not necessary to link station selection to cases that had been reported. In any case, majority of the reported cases were from the GAR but had not been disaggregated by location.
